# Casein‐enhanced uptake and disease‐modifying bioactivity of ingested extracellular vesicles

**DOI:** 10.1002/jev2.12045

**Published:** 2021-01-11

**Authors:** Mark A. Aminzadeh, Mario Fournier, Akbarshakh Akhmerov, K. Candis Jones‐Ungerleider, Jackelyn B. Valle, Eduardo Marbán

**Affiliations:** ^1^ Cedars‐Sinai Medical Center Smidt Heart Institute Los Angeles California USA

**Keywords:** casein, extracellular vesicles, ingestion

## Abstract

Extracellular vesicles (EVs) from cardiac stromal cells, developed as therapeutic candidates, improve dystrophic muscle function when administered parenterally, but oral delivery remains untested. We find that casein, the dominant protein in breast milk, enhances the uptake and bioactivity of ingested heart‐derived EVs, altering gene expression in blood cells and enhancing muscle function in *mdx* mice with muscular dystrophy. Thus, EVs, administered orally, are absorbed and exert disease‐modifying bioactivity *in vivo*. Formulating EVs with casein enhances uptake and markedly expands the range of potential therapeutic applications.

## 1 INTRODUCTION

Extracellular vesicles (EVs) are plentiful in all body fluids, including milk (de la Torre Gomez et al., 2018). These vesicles are packed with bioactive RNAs, including microRNAs and other noncoding RNAs, which can alter gene expression in target cells (Zhou et al., 2012). EVs may confer many of the benefits associated with breast milk (Admyre et al., 2007; de la Torre Gomez et al., 2018; Hock et al., 2017), but this conjecture is challenged by the harsh conditions of gastrointestinal digestion (Liao et al., 2017). Evolutionarily, the proteins in milk have structural disorderedness and micelle‐forming capabilities which make them, and enclosed bioactive factors, resistant to extreme environments (Gigli, 2016). Thus, we hypothesized that admixture with milk proteins would enhance gastrointestinal uptake and systemic bioactivity of ingested EVs. To test this idea, we used EVs secreted by human cardiosphere‐derived stromal/progenitor cells (Aminzadeh et al., 2018; Ibrahim et al., 2014; Marban, 2018) (CDC‐EV), and casein, the predominant protein component of milk. CDC‐EV, when delivered parenterally, strikingly benefit cardiac and skeletal muscle function in *mdx* mice (Aminzadeh et al., 2018; Rogers et al., 2019), a model of Duchenne muscular dystrophy, but the effects of oral CDC‐EV are unknown.

## 2 MATERIALS AND METHODS

See Supplemental Information for detailed methods. We have submitted all relevant data to the EV‐TRACK knowledgebase (EV‐TRACK ID: EV200125) (EV‐TRACK Consortium et al., 2017).

### 2.1 Statistical analysis

All results are presented as mean ± SEM. Normality and equality of variances of data sets were first tested using Kolmogorov‐Smirnov and Levene's tests, respectively. If both were confirmed, t‐test was used for determination of statistical significance; if either normality or equality of variances was not assured, nonparametric tests (Wilcoxon test or Kruskal‐Wallis test followed by Dunn's post‐test) were applied (SPSS II, SPSS Inc., Chicago, Illinois, USA). No preliminary data were available for a power analysis. Results from the pilot project allowed us to power subsequent studies.

### 2.2 Animal studies

All animals were housed in a temperature‐controlled environment with a 12‐h light cycle, given standard chow diet and water ad libitum, and handled according to the Animal Care and Use Committee of Cedars Sinai Medical Center, under an approved protocol (IACUC4545). For all experiments, age‐ and gender‐matched wild‐type or *mdx* mice (2–12 months) were used.

### 2.3 CDC‐EV

EVs were isolated from plasma‐free media conditioned overnight (24 h) by cultured human CDCs using ultracentrifugation (Aminzadeh et al., 2018). EVs were counted using the NanoSight NS300 system (NanoSight Ltd, UK) (Aminzadeh et al., 2018) and 10^7^ EVs were mixed with 8% casein solution which was made in PBS. The mixture of casein solution and CDC‐EV was fed to *mdx* mice by oral gavage after 18 h of only‐food fasting. Other experimental *mdx* mice were fed with 10^7^ EVs in PBS, PBS alone or 8% casein solution alone after 18 h of only‐food fasting. EVs were gold‐labelled using gold‐conjugated anti‐CD63 antibody and were imaged in *mdx* mice duodenal sections by transmission electron microscopy and tomography about 10 min after oral ingestion of EVs. In order to determine presence of human‐specific RNAs from CDC‐EV in *mdx* mouse plasma after oral administration, RNA reads shared in casein, *mdx* mouse plasma and human EVs were excluded and only RNAs which were specific to human EVs were considered as biomarkers to assess gut absorption of EVs. One hour after oral administration of human CDC‐EV with and without casein, mice were sacrificed, and blood was collected from the inferior vena cava for RNA sequencing and fluorescence‐activated cell sorting. Biodistribution of EVs was assessed by measuring elemental gold within tissue. About 10 min after oral ingestion of immuno‐gold labelled CDC‐EV, or gold particles with casein (as control for CDC‐EV), or PBS alone (as control for all interventions), mice were sacrificed and plasma and 50 mg of tissues from different organs were subjected to Neutron Activation Analysis (Figure S1A).

### 2.4 Phenotyping

Echocardiographic studies were performed 2 days before (Baseline) and 3 weeks after every other day oral delivery of EVs with and without casein using the Vevo Imaging System (VisualSonics, Toronto, Canada) described previously (Aminzadeh et al., 2018). In vitro isometric contractile properties of soleus muscle and exercise capacity were assessed 3 weeks after every other day administration of EVs with and without casein using the methods described previously (Aminzadeh et al., 2018).

## 3 RESULTS

### 3.1 Transmission electron microscopy of casein micelles with CDC EVs

**FIGURE 1:**
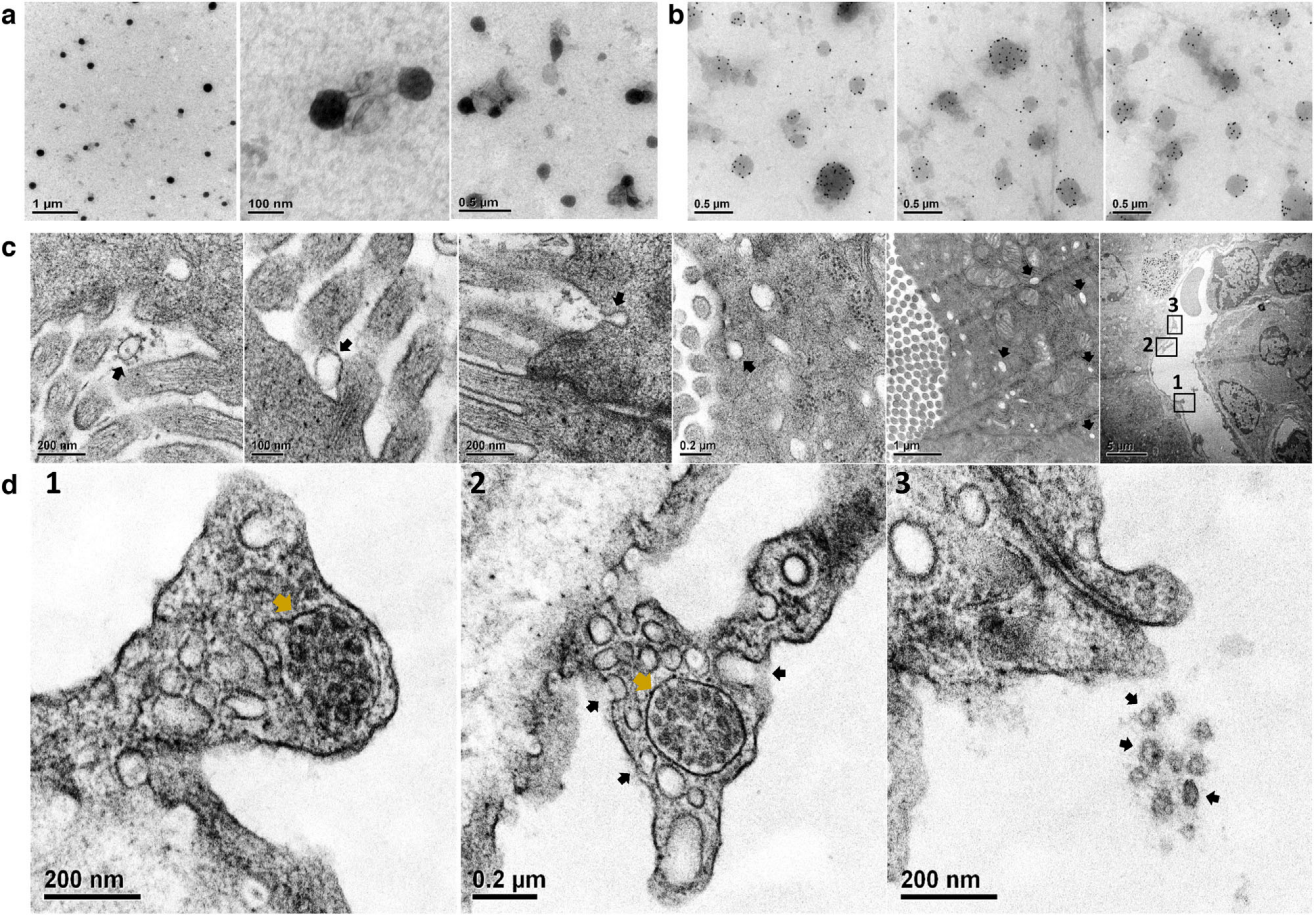
Transmission electron microscopy (TEM) of casein micelles with CDC‐EV (a) immuno‐gold labelled CDC‐EV (b) and *mdx* mouse duodenum ∼10 min after oral gavage delivery of immuno‐gold labelled CDC‐EV with casein (c and d). Immuno‐gold labelled CDC‐EV were pointed with black arrows in the duodenal lumen, at and inside duodenal epithelia cell and inside duodenal vessel (c). D1‐3: Enlarged TEM images of a duodenal vessel (row C last image) showing immuno‐gold labelled CDC‐EV in a multi vesicular body next to vessel wall (MVB, d1, d2 gold arrows), immuno‐gold labelled CDC‐EV fusing with vessel wall (d2, black arrows) and, intravascular immuno‐gold labelled CDC‐EV (d3, black arrows)

**FIGURE 2:**
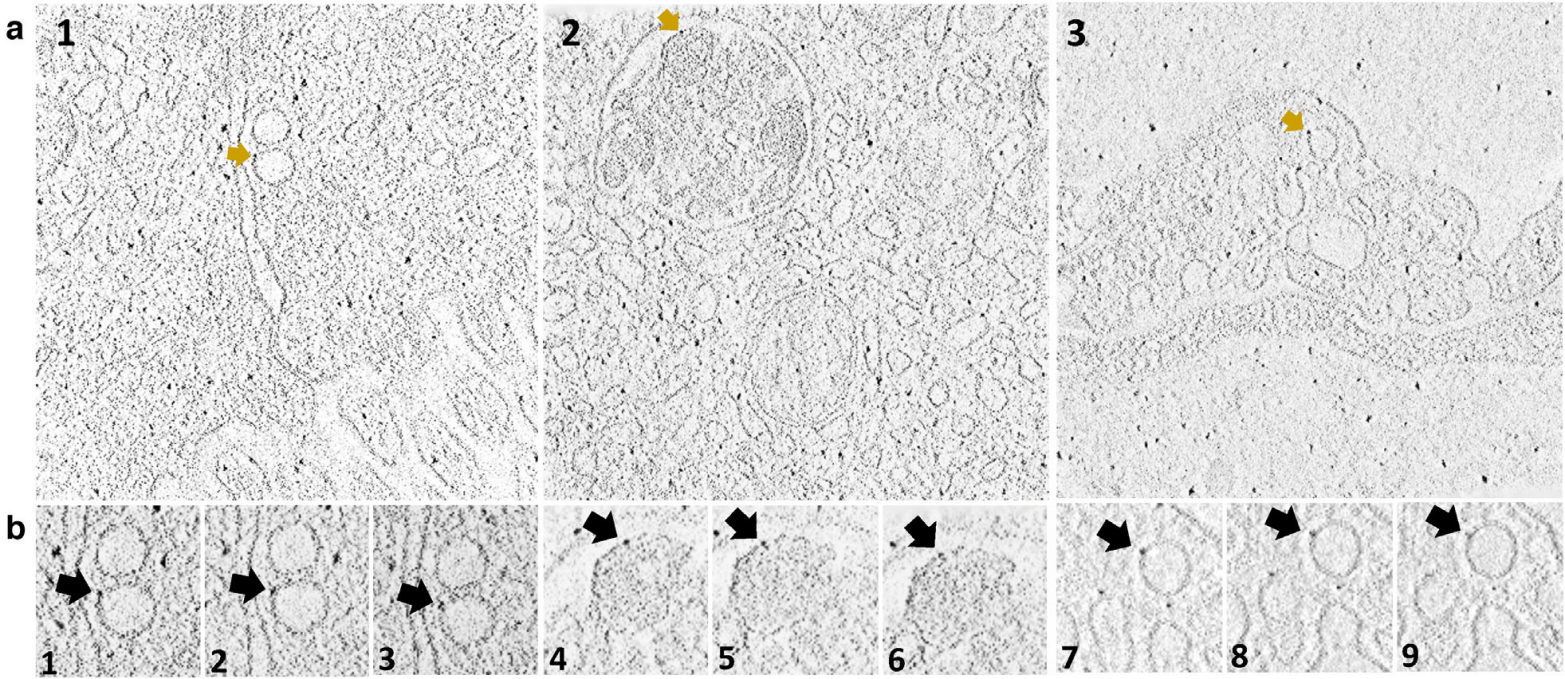
(a and b) 3D tomography of immuno‐gold labelled CDC‐EV in duodenal epithelial cell cytoplasm (a1), inside a multi vesicular body (a2) and inside duodenal vascular endothelia cell (a3). (b) Adjacent sequential optical slices of a1 (b1, 2 and 3), a2 (b4, 5 and 6) and a3 (b7, 8 and 9) demonstrating that gold particles are attached to CDC‐EV membrane

Transmission electron microscopy revealed that CDC‐EV mixed with casein (CDC‐EV+C) were encapsulated within micelles (Figure 1a), which may help protect from the harsh digestive environment. To localize and assess fate after ingestion, CDC‐EV were immuno‐gold labelled (Figure 1b), mixed with casein and fed to *mdx* mice. Ten minutes after oral delivery, labelled CDC‐EV could be seen in the duodenal lumen, inside epithelial cells and even within the microvasculature (Figures 1c and 1D1‐3). Sequential tomography further confirmed the transepithelial distribution of immunogold‐labelled CDC‐EV after oral delivery (Figures 2a and 2b).

### 3.2 Biodistribution of CDC‐EV after ingestion

By neutron activation analysis using gamma ray spectroscopy, gold particles were evident in tissues of *mdx* mice fed with gold‐labelled CDC‐EV mixed with casein but not in mice fed with only gold mixed with casein. Just 10 min after ingestion of immunogold‐labelled CDC‐EV, gold was detectable in plasma, diaphragm, soleus and tibialis anterior muscles (Figure S1A), stringently demonstrating CDC‐EV uptake after oral delivery.

### 3.3 Human‐specific RNAs from EVs

To detect RNAs from human CDC‐EVs in *mdx* mouse plasma after CDC‐EV ingestion, we extracted and sequenced RNAs from casein proteins and also control *mdx* mouse plasma and excluded RNA reads from CDC‐EVs which were found also in casein protein and/or *mdx* mouse plasma. We called the non‐excluded RNAs ‘human‐specific RNA from CDC‐EV’ (Figure S1B). One hour after ingestion of CDC‐EV+C, 99% of human‐specific RNAs from EVs (Figure S1C) could be detected in *mdx* mouse plasma by next‐generation RNA sequencing. After ingestion of CDC‐EV without casein, only 16% of human‐specific RNAs from EVs could be detected in mouse plasma (Figure S1C). Copy numbers of human‐specific RNAs from EVs were also higher in plasma of *mdx* mice fed with CDC‐EV+C compared to *mdx* mice fed CDC‐EV without casein (Figure S1D).

### 3.4 Whole blood RNA expression and cell population after EV ingestion

**FIGURE 3:**
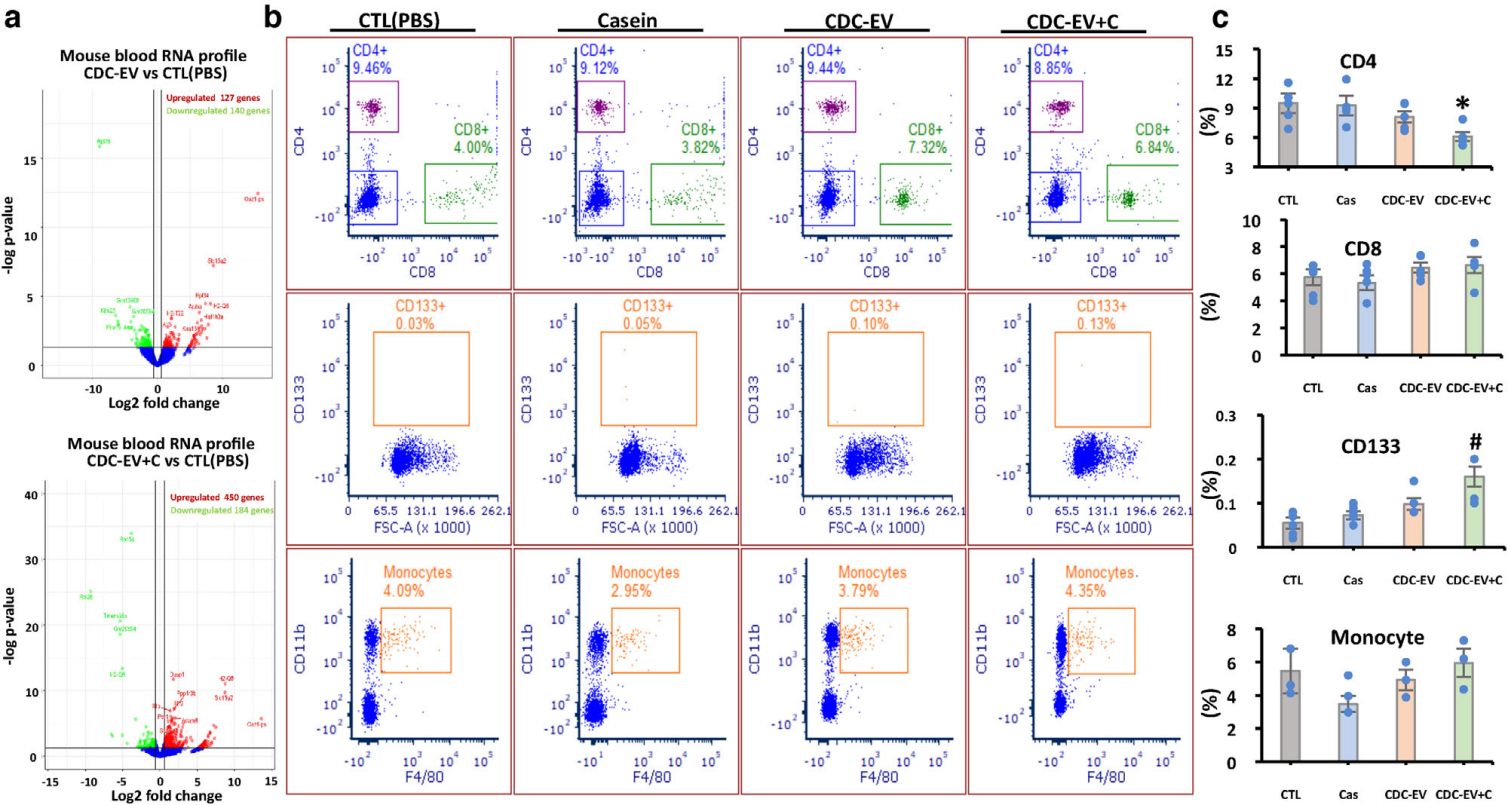
Whole blood RNA expression (a) and cell population (b) and their percentages 1 h after ingestion of PBS (CTL), casein alone (Cas), CDC‐EV or CDC‐EV with casein (CDC‐EV+C). Data are mean + SEM; *n* = 3–5 in each group. *P < 0.05 vs CTL, Cas or CDC‐EV; *^#^P <* 0.015 vs CTL or Cas

Ingestion of human CDC‐EV altered gene expression. The volcano plots in Figure 3a show major changes in whole blood RNA expression with CDC‐EV, but even greater changes with CDC‐EV+C (Figure 3a). The percentages of CD4^+^ T cells and CD133^+^ cells were likewise more prominently altered in (CDC‐EV+C)‐treated *mdx* mice relative to *mdx* mice which had received only CDC‐EV (Figure 3b,c).

### 3.5 Cardiac ejection fraction, soleus muscle specific force and exercise capacity

**FIGURE 4:**
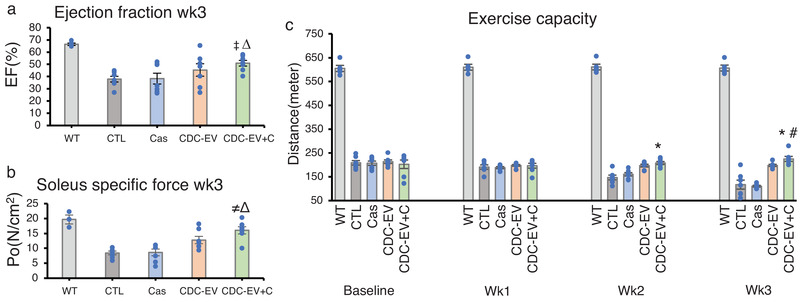
Cardiac ejection fraction (a), soleus muscle specific force (b) and exercise capacity (c) of mdx mice fed every other day for 3 weeks with vehicle only (PBS(CTL)), casein alone (Cas), CDC‐EV, or CDC‐EV mixed with casein (CDC‐EV+C). Wild type mice (WT) served as control. Data are means + SEM; *n* = 5–7 in each group Δ*P *< 0.005 vs CTL; *^‡^P *< 0.05 vs CDC‐EV; *^≠^P* < 0.005 vs CDC‐EV; ^*^P < 0.005 vs CTL or Cas; ^#^ *P *< 0.01 vs CDC‐EV

Duchenne muscular dystrophy is a genetic disease marked by a skeletal myopathy and a cardiomyopathy, both of which can be modelled in *mdx* mice (Aminzadeh et al., 2018). To assess disease‐modifying bioactivity, every other day we fed *mdx* mice PBS, casein, CDC‐EV or CDC‐EV+C and measured function of the heart and skeletal muscle. Casein itself had no benefits, but, after 3 weeks, mice fed CDC‐EV+C exhibited a distinct increase in ejection fraction, a global measure of heart function, which was greater than that elicited by CDC‐EV alone (Figure 4a). Skeletal muscle function likewise improved with oral CDC‐EV+C, whether assessed ex vivo by specific force of the isolated soleus (Figure 4b) or in vivo by ambulatory capacity (Figure 4c). Although CDC‐EV was somewhat effective, CDC‐EV+C exhibited significantly higher improvements in all measures, which highlights the nonlinear relationship between EV dose bioavailability and functional benefit. Based on previous studies (Rogers et al., 2019), the benefits seen with CDC‐EV+C are nearly identical with those of intravenous CDC‐EV, hinting that the benefit may be maximal. Thus, oral CDC‐EVs appear to be sufficiently absorbed to achieve nearly‐maximal benefit.

## 4 DISCUSSION

EVs are abundant in various foods, notably beer, milk and plant juices (Xiao et al., 2018). Rampant speculation has centred around the potential roles of food EVs, both in nutrition and in nutraceutical applications, but a paucity of solid evidence has been compounded by controversy (Zempleni, 2017). For example, a claim that fluorescently‐labelled bovine milk EVs can be detected in virtually all peripheral tissues of rodents was based on studies lacking vehicle controls (Agrawal et al., 2017). Likewise, findings that microRNAs in milk EVs traverse the gastrointestinal barrier to appear in host blood, exerting systemic bioactivity across species boundaries, have been criticized on technical grounds including sample contamination (Zempleni et al., 2017). Here, we have demonstrated that ingested EVs are indeed bioactive in the host, especially after EVs are mixed with casein. Breast milk contains copious amounts of EVs which have been postulated to confer immune benefits (Admyre et al., 2007; Hock et al., 2017); if so, it is logical that the proteins in breast milk would enhance EV uptake. Casein phosphoproteins (αS1, αS2, β, and κ casein) comprising about 40–80% of the protein in breast milk exhibit a disordered conformation with chaperone activity (αS1, β), enabling self‐association and assembly into functional micelles which can encapsulate other substances (Gigli, 2016). In fact, our study demonstrated CDC‐EV mixed with casein are encapsulated within micelles and are more broadly biodistributed after ingestion. More generally, our work bolsters the concept of meaningful oral EV uptake, based on the following evidence: visualization of immunogold‐labelled CDC‐EV traversing the gastrointestinal barrier by transmission electron microscopy; detection of elemental gold in plasma and various host muscles after ingestion; documentation of changes in gene expression in peripheral blood after CDC‐EV ingestion; and evidence of disease‐modifying bioactivity when CDC‐EV are delivered orally to *mdx* mice, particularly in combination with casein. The findings open up the prospect of consistent and effective delivery of EVs, a versatile new class of therapeutic agents, via simple ingestion.

## CONFLICTS OF INTEREST

EM declares ownership of founder's equity in Capricor Therapeutics. Other authors declare no conflict of interest.

## Supporting information



Supporting information

## Data Availability

We have submitted all relevant data of our experiments to the EV‐TRACK knowledgebase (EV‐TRACK ID: EV200125) (Van Deun J, et al. EV‐TRACK: transparent reporting and centralizing knowledge in extracellular vesicle research. Nature methods. 2017; 14(3):228‐32). You may access and check the submission of experimental parameters to the EV‐TRACK knowledgebase via the following URL: http://evtrack.org/review.php. Please use the EV‐TRACK ID (EV200125) and the last name of the first author (Aminzadeh) to access our submission.
