# Supplementary material for: Casein‐enhanced uptake and disease‐modifying bioactivity of ingested extracellular vesicles
Source: J Extracell Vesicles. 2021 Jan 11;10(3):e12045. doi: 10.1002/jev2.12045 (PMC7798403; doi:10.1002/jev2.12045)
Supplement: Supplementary file 1 — Supporting information [file JEV2-10-e12045-s001.docx]

**Detailed Methods:**

**CDC-EV:** EVs were isolated from serum-free media conditioned overnight (24 hr) by cultured human male CDCs in hypoxia (2% O2) as described previously (1). Ultracentrifugation (100,000 x g for 1 hr) was used to isolate EVs from conditioned media after sequential centrifugations at 300 x g (10 min) and 10,000 x g (30 min) and filtration with 0.22 mm filters (2). Isolated EVs were re-suspended in PBS and were analyzed by nanoparticle tracking, using the NanoSight NS300 system (NanoSight Ltd, UK). Videos were collected and analyzed using NTAsoftware (version 2.3), with the minimal expected particle size, minimum track length, and blur setting all set to automatic. Camera shutter speed was fixed at 30.01 ms and camera gain was set to 500. Camera sensitivity and detection threshold were set close to maximum (15 or 16) and minimum (3 or 4), respectively, to reveal small particles. Ambient temperature was recorded manually, ranging from 24 to 27°C. For each sample, five videos of 60 seconds duration were recorded, with a 10-second delay between recordings, generating five replicate histograms that were averaged. Size of CDC-EVs was 50 to 400nm; 150nm EVs comprised the highest number of EVs. This observation was consistent with our previous study (1). The ratio of CDC-EV to protein in re-suspended isolated CDC-EV in PBS was also measured. Protein concentration was measured using Micro BCA Protein Assay Kit (Life Technologies, Grand Island, NY). In our previous work we characterized identically-prepared CDC-EVs using western blotting. Preliminary dose-ranging experiments with oral ingestion of 10^7^, 5x10^7^ and 50x10^7^ CDC-EVs revealed the lowest dose sufficed to induce the described changes in whole blood pathways. CDC-EV were characterized by the most rigorous of criteria as described previously (1). The vesicles are variable in size and morphology, consistent with previous work (1). Isolated CDC-EV were admixtured in 300 microliter of PBS or 8% casein solution and were given orally to *mdx* mice by oral gavage. **Casein**: Micellar Casein protein, Lab Grade, was purchased from Beyond Raw Chemistry Labs (BR0013, PA); 8% casein solution was made in PBS. **Immuno-gold labeled CDC-EV**: 10^7^ CDC-EV were immune-gold labeled using gold conjugated CD63 antibody (concentration~600 gold nanoparticles/CDC-EV; Nanopartz: CA11-20-ACD63-MH-DIH-250-1, CO, USA) and were then given orally by oral gavage in 300 microliter of 8% casein solution. **Electron microscopy**: 3 cubes from anterior and posterior parts of duodenum from each *mdx* mice (CDC-EV: n=3; CDC-EV+C: n=3) were fixed by immersion of 1mm^3^ cubes in 3% glutaraldehyde, postfixed in osmium, and embedded in epon. Sections were cut at silver thickness, stained with uranyl acetate and lead citrate, and viewed with JEOL 1010 equipped with AMT digital camera system (Figure 1C&D) and with TF20 high resolution EM for tomography and 3D imaging (Figure 2A&B). EM was performed at California NanoSystem Institute at UCLA. **Biodistribution**: Biodistribution of CDC-EV was assed using Neutron activation analysis. About 10 minutes after oral ingestion of immuno-gold labeled CDC-EV, gold particles with casein (served as control) or PBS alone (control mice), mice were sacrificed and plasma and 50 mg of tissues from different organs were used for Neutron Activation Analysis. Neutron irradiation was done at the research reactor, University of Texas at Austin. After sufficient decay, gamma ray spectroscopy was done to measure 411 keV characteristic Au gamma ray, and quantification by using Au standard (Supplemental Figure 1A; Cerium Laboratories, Austin, Tx). **Flowcytometry:** Whole blood collected from inferior vena cava vein by venipuncture in heparin-treated collection tubes. 1mL heparin-treated whole blood and 10mL RBC lysis buffer (Gibco™ ACK Lysing Buffer; cat#A1049201) were mixed, incubated for 3-5 minutes and then, centrifugated for 5 minutes at 300 x g at room temperature. Supernatant was aspirated, leaving approximately 50 uL to avoid disturbing the pellet. 5 mL cold phosphate buffered saline was added and then gently the cells and the remaining fluid were mixed and again re-centrifugated at 300 x g for 5 minutes at 2-8°C. The supernatant was aspirated and then the cells resuspend in FACS buffer (PBS containing 1% FBS and 2 mM EDTA) and stained with conjugated antibody for 20 to 30 minutes at 4°C. Cells were washed and resuspended with FACS buffer for flow cytometric analyses using the following antibodies: PE Rat Anti-Mouse CD8a (Clone 53-6.7, Cat# 553036, BD Biosciences), Alexa Fluo 8 ® 488 Rat Anti-Mouse CD4 (Clone RM 4–5, Cat# 557667, BD Biosciences), APC Anti-Mouse CD133 (Clone 13A4, Cat#17-1331-81, Life Technologies) and PE Anti-Mouse F4/80 (Clone BM8, Cat#12-4801-80, Life Technologies).

**Animal study:** we studied female *mdx* mice with spontaneous *Dmd^mdx^* mutation which do not express dystrophin [C57BL/10ScSn-*Dmd^mdx^*/J] and have Duchene muscular dystrophy phenotype, as described previously(1). Female C57BL/10ScSnJ mice served as wild type control (Jackson Laboratory, USA). 8-week-old *mdx* mice were randomized to PBS, casein, CDC-EV or CDC-EV+C, fasted for 18 hours (water available) and were sacrificed 10 and 60 minutes after oral ingestion of 300 microliter of PBS, casein (8% solution in PBS), CDC-EV (10^7^ CDC-EV in PBS) and CDC-EV+C (10^7^ EV in casein solution) for studies on TEM, bio-distribution, blood/plasma RNA profiling and blood flow cytometry. 10 minutes after oral ingestion of immuno-gold labeled CDC-EV+C (10^7^ immuno-gold labeled CDC-EV mixed with 8% casein solution in PBS), *mdx* mice (n=3) were sacrificed and 0.2x0.2 cm full thickness duodenum were dissected and fixed in 3% glutaraldehyde for TEM studies. Tissue preparation including plastic embedding for TEM studies were performed at California NanoSystem Institute at UCLA. 60 minutes after ingestion of PBS (n=3), casein (n=3), CDC-EV (n=3) or CDC-EV+C (n=3), *mdx* mice were sacrificed and blood from each experimental group were collected, mixed and used as a single sample for blood RNA profiling using next generation RNA sequencing (Figure 3A; UCLA Technology Center for Genomic & Bioinformatics). Similar separate experiment was performed for plasma collection from blood (n=3 for each experimental group) and for plasma RNA profiling using next generation RNA sequencing (Supplemental Figure 1 C&D; UCLA Technology Center for Genomic & Bioinformatics). Study on cell populations in blood (60 minutes after oral ingestion of PBS, casein, CDC-EV, CDC-EV+C) followed the same experimental design (n=3 in each experimental group; blood from each experimental group was collected, mixed and was used as a single sample for flow cytometry) using flow cytometry (Cedars-Sinai Flow Cytometry Core). Data presented in Figure 3C depict 5 experimental repeats for each group PBS, casein, CDC-EV and CDC-EV+C and 3 experimental repeats for wild type control group. For evaluation of cardiac function, contractile properties of soleus muscle and exercise capacity, 12-month-old *mdx* mice were randomized to PBS (n=7), casein (n=7), CDC-EV (n=7) or CDC-EV+C (n=7) and were fed every other day for 3 weeks. After 3 weeks *mdx* mice were initially evaluated for cardiac function using echocardiography and then (the day after echocardiography) assessed for exercise capacity applying treadmill and ultimately (same day as exercise capacity was evaluated) were sacrificed for soleus muscle dissection and ex-vivo evaluation of soleus muscle contractile properties.

**RNA sequencing and 2-Dimensional hierarchical clustering:** The KAPA RNA HyperPrep Kits (Kapa Biosystems, Wilmington MA) was used to make the sequencing library. The workflow consists of rRNA depletion, RNA fragmentation, double strand cDNA generation, A-tailing, adaptor ligation and PCR amplification. Different adaptors were used for multiplexing samples in one lane. Sequencing was performed on Illumina HiSeq 3000 for a pair read 150 run. Data quality check was done on Illumina SAV. Demultiplexing was performed with Illumina bcl2fastq. The reads were first mapped to the latest UCSC transcript set using Bowtie2 version 2.1.0 and the gene expression level was estimated using RSEM v1.2.15. TMM (trimmed mean of M-values) was used to normalize the gene expression. Differentially expressed genes were identified using the edgeR program. Genes showing altered expression with p<0.05 and more than 2 fold changes were considered differentially expressed. The R package ggplot2 was used for the volcano plot showing the statistical significance (P value) versus magnitude of change (fold change). The significantly up-regulated genes were colored in red and down-regulated genes were colored in green (Figure 3A and Supplemental Figures 1C&D).

**Echocardiography:** Echocardiographic studies were performed two days before (Baseline) and 3 weeks after every other day oral delivery of CDC-EV with and without casein using the Vevo Imaging System (VisualSonics, Toronto, Canada) described previously (1). After induction of light general anesthesia (1.5% inhaled isoflurane), the heart was imaged at the level of the greatest LV diameter. LV ejection fraction (LVEF) was measured with Vevo Lab software 2.1.0 from 2-dimensional long-axis views and were obtained by averaging three measurements from each animal. Echocardiographic studies were read independently by investigator blinded to treatment allocation.

**In vitro isometric contractile properties of soleus muscle:** Mice were deeply anesthetized with isoflurane, the soleus (SOL) muscles were rapidly excised, and the animals euthanized. Briefly, following a lateral midline skin incision of the lower leg the SOL muscle was dissected and isolated and its tendons of origin and insertion were tightened with silk suture (3-0) and rapidly excised. The SOL muscle was vertically mounted in a tissue bath (Radnoti, Covina CA) containing a mammalian Ringer's solution of the following composition: (in mM) 137 NaCl, 5 KCl, 2 CaCl2, 1 MgSO4, 1 NaH2PO4, 24 NaHCO3, 11 glucose. The solution was constantly aerated with 95% O2 and 5% CO2 with pH maintained at 7.35 and temperature kept at 24°C. One end of the SOL was secured to a clamp at the bottom of the dish and one end was attached to a calibrated force transducer (Aurora Scientific, Model 300C; Aurora, ON, Canada). A micromanipulator linked to the system was used to adjust muscle length. Platinum plate electrodes placed on each side of the muscle were used for direct muscle stimulation using 0.2 msec duration monophasic rectangular pulses of constant current delivered at supramaximal intensity. An Aurora Scientific 610A Dynamic Muscle Control Suite v5.510 was used to conduct the experiment. Muscle length was adjusted until maximum isometric twitch force responses were obtained. Isometric contractile properties were determined at this optimal length (Lo) measured with a digital caliper (Mitutoya, Kanagawa, Japan). Peak twitch force (Pt) was determined from a series of single pulses. Force/frequency relationships were measured at stimulus frequencies ranging from 5-180 pulses per second (pps). The stimuli were presented in trains of 1 sec duration with an interval of 1 min intervening between each stimulus train. Recordings of muscle contractions were analyzed using an Aurora Scientific 611A Dynamic Muscle Analysis software v5.321. All SOL muscle forces generated, including Pt and maximum tetanic force (Po), were normalized for the estimated physiological cross-sectional areas (CSA) of the muscle (CSA = muscle weight/1.056 x 0.71 x Lo; where 1.056 g/cm3 represents the density of muscle and 0.71 is the ratio of muscle fiber length to muscle Lo) and expressed in Newtons (N)/cm2. Absolute SOL muscle forces were also reported in mN, as previously described (1).

**Treadmill exercise testing:** For Figure 4C, exercise capacity was assessed weekly with Exer-3/6 open treadmill (Columbus Instruments, Columbus, OH), beginning 1 week prior to initiation of oral gavage administration of PBS, casein, CDC-EV and CDC-EV+C and 3 weeks after. After an acclimation period (10 m/min for 20 min), stepwise increases in average speed (2 m/min) were applied every two minutes during treadmill exercise until the mouse became exhausted (spending >10 seconds on shocker; continuous nudging was used during treadmill to help mice stay on the track). Subsequently, the mouse was returned to the cage and the total distance recorded. The treadmill protocol conformed to guidelines from the American Physiological Society described previously (1).


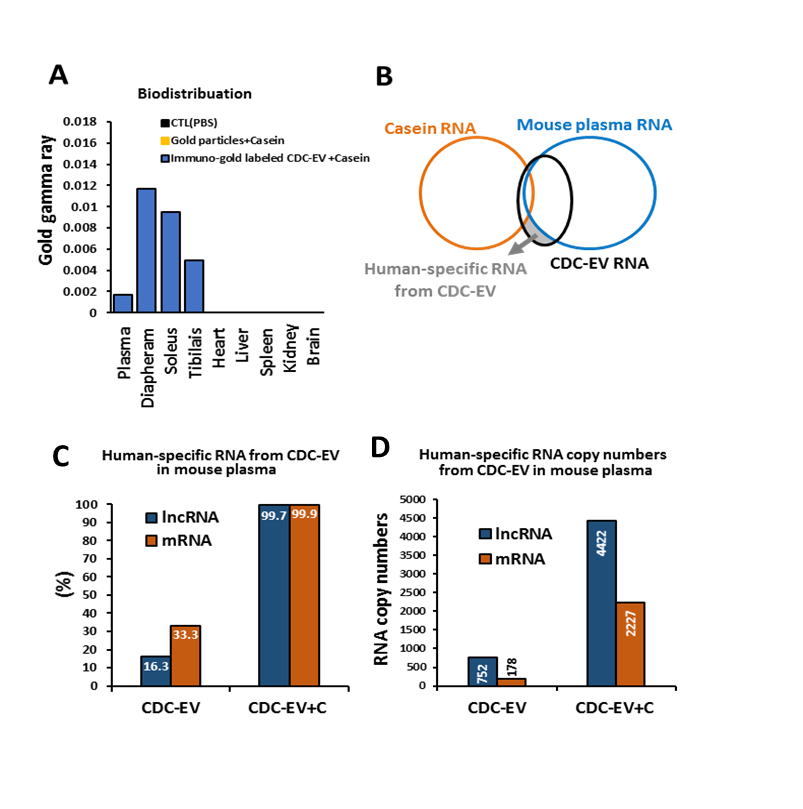


**Supplemental figure 1A-D**: **A:** Biodistribution of immuno-gold labeled CDC-EV in different *mdx* mouse organs 10 minutes after ingestion of CDC-EV with casein. **B:** Schematic figure showing human-specific RNA from CDC-EV (gray area) which are not found in *mdx* mouse plasma and bovine casein proteins. **C&D:** Percentage of human-specific RNA from CDC-EV (**C**) and their copy numbers (**D**) in *mdx* mouse plasma 1 hour after ingestion of human CDC-EV with (CDC-EV+C) and without (CDC-EV) casein.

**References:**

1. Aminzadeh MA, Rogers RG, Fournier M, Tobin RE, Guan X, Childers MK, Andres AM, Taylor DJ, Ibrahim A, Ding X, et al. CDC-EVsome-Mediated Benefits of Cell Therapy in Mouse and Human Models of Duchenne Muscular Dystrophy. *Stem Cell Reports.* 2018;10(3):942-55.

2. Lasser C, Eldh M, and Lotvall J. Isolation and characterization of RNA-containing. *J Vis Exp.* 201259):e3037.
